# Minimal clinically important decline in physical function over one year: EPOSA study

**DOI:** 10.1186/s12891-019-2593-1

**Published:** 2019-05-17

**Authors:** Paola Siviero, Federica Limongi, Antonella Gesmundo, Sabina Zambon, Cyrus Cooper, Elaine M. Dennison, Mark H. Edwards, Erik J. Timmermans, Suzan van der Pas, Laura A. Schaap, Natasja M. van Schoor, Michael D. Denkinger, Florian Herbolsheimer, Richard Peter, Maria Victoria Castell, Ángel Otero, Rocio Queipo, Nancy L. Pedersen, Dorly J. H. Deeg, Stefania Maggi, for the EPOSA Research Group, T. Nikolaus, R. Peter, M. D. Denkinger, F. Herbolsheimer, S. Maggi, S. Zambon, F. Limongi, M. Noale, P. Siviero, D. J. Deeg, S. van der Pas, L. A. Schaap, N. M. van Schoor, E. J. Timmermans, A. Otero, M. V. Castell, M. Sanchez-Martinez, R. Quieipo, N. L. Pedersen, R. Broumandi, E. M. Dennison, C. Cooper, M. H. Edwards, C. Parsons

**Affiliations:** 1 National Research Council, Neuroscience Institute - Aging Branch, Via Giustiniani 2, ,35128 Padova, Italy; 2 Department of Medicine, University of Padova, Via 8 Febbraio 2, 35122 Padova, Italy; 3 MRC Lifecourse Epidemiology Unit, University of Southampton, Southampton General Hospital, Tremona Road, Southampton, SO16 6YD UK; 4 Portsmouth Hospitals NHS Trust, Portsmouth, PO6 3LY UK; 5 Department of Epidemiology and Biostatistics, Amsterdam UMC - location VU University medical center, Amsterdam Public Health research institute, Amsterdam, the Netherlands; 6 Bethesda Geriatric Clinic, University of Ulm, Zollernring 26, 89073 Ulm, Germany; 7 Institute of the History Philosophy and Ethics of Medicine, University of Ulm, Frauensteige 6, 89075 Ulm, Germany; 8 Department of Preventive Medicine and Public Health, Unit of Primary Care and Family Medicine, Faculty of Medicine, Universidad Autonoma de Madrid, Arzobispo Morcillo 4, 28029 Madrid, Spain; 9 Department of Medical Epidemiology and Biostatistics, Karolinska Institutet, P.O.Box 281, Nobels väg 12A, SE-171 77 Stockholm, Sweden

**Keywords:** Osteoarthritis, MCID, Functional decline, Hand, Hip/knee, AUSCAN, WOMAC

## Abstract

**Background:**

The Australian/Canadian hand Osteoarthritis Index (AUSCAN) and the Western Ontario and McMaster Universities knee and hip Osteoarthritis Index (WOMAC) are the most commonly used clinical tools to manage and monitor osteoarthritis (OA). Few studies have as yet reported longitudinal changes in the AUSCAN index regarding the hand. While there are published data regarding WOMAC assessments of the hip and the knee, the two sites have always evaluated separately. The current study therefore sought to determine the minimal clinically important difference (MCID) in decline in the AUSCAN hand and WOMAC hip/knee physical function scores over 1 year using anchor-based and distribution-based methods.

**Methods:**

The study analysed data collected by the European Project on Osteoarthritis, a prospective observational study investigating six adult cohorts with and without OA by evaluating changes in the AUSCAN and WOMAC physical function scores at baseline and 12–18 months later. Pain and stiffness scores, the performance-based grip strength and walking speed and health-related quality of life measures were used as the study’s anchors. Receiver operating characteristic curves and distribution-based methods were used to estimate the MCID in the AUSCAN and WOMAC physical function scores; only the data of those participants who possessed paired (baseline and follow up-measures) AUSCAN and WOMAC scores were included in the analysis.

**Results:**

Out of the 1866 participants who were evaluated, 1842 had paired AUSCAN scores and 1845 had paired WOMAC scores. The changes in the AUSCAN physical function score correlated significantly with those in the AUSCAN pain score (*r* = 0.31). Anchor- and distribution-based approaches converged identifying 4 as the MCID for decline in the AUSCAN hand physical function. Changes in the WOMAC hip/knee physical function score were significantly correlated with changes in both the WOMAC pain score (*r* = 0.47) and the WOMAC stiffness score (*r* = 0.35). The different approaches converged identifying two as the MCID for decline in the WOMAC hip/knee physical function.

**Conclusions:**

The most reliable MCID estimates of decline over 1 year in the AUSCAN hand and WOMAC hip/knee physical function scores were 4 and 2 points, respectively.

## Background

The Australian/Canadian Hand Osteoarthritis Index (AUSCAN) [1] and the Western Ontario and McMaster Universities Osteoarthritis Index (WOMAC) [2, 3] scales are self-report instruments measuring pain, stiffness and physical function linked to osteoarthritis (OA), and have been used by the European Project on Osteoarthritis (EPOSA) to assess personal and societal variables affected by OA, such as quality of life (QoL), social participation, and health care use in several ageing European cohorts. The individuals enrolled in the project were receiving treatment for severe OA, had undiagnosed or untreated OA or did not have OA at all [4].

The Minimum Clinically Important Difference (MCID) is defined as the smallest change in a score that a patient perceives as beneficial or detrimental [5]. There are different types of MCID, depending on whether there has been an improvement or a worsening in the variable being measured and on the external standard being employed [6].

Until now, to our knowledge, the MCID in the AUSCAN hand and WOMAC hip/knee physical function scales has received scarce attention. Specifically few studies report longitudinal changes in the AUSCAN [7]. While some studies have investigated the WOMAC scales [7–9], the two sites of hip and knee have always been evaluated separately [10–15]. Moreover, the MCID has almost always been considered from an improvement perspective, as the majority of studies have aimed to examine the efficacy of pharmacological interventions [11, 13], rehabilitation programs [8, 9], and/or of surgical treatments [10, 12, 14, 15].

The aim of the current study was therefore to estimate the MCID in the AUSCAN and WOMAC physical function subscales using distribution-based and anchor-based methods for longitudinal changes. We postulated that the AUSCAN and the WOMAC physical function scores would worsen [16, 17] with time (i.e., there would be a rise in both) and that the changes in the AUSCAN and WOMAC physical function scores would correlate significantly with changes in other well-established OA health variables or performance-based measures [18–25].

## Methods

### Participants

The current study analysed data collected by the European Project on OSteoArthritis (EPOSA), a population-based study involving cohorts living in Germany, Italy, the Netherlands, Spain, Sweden, and the UK, that recruited 2942 adults between the ages of 65–85. All the participants gave written informed consent; the study design and methodology are outlined in detail elsewhere [4]. The study design was granted approval by the appropriate local ethics committees (Germany: Universitat Ulm Ethikkommission [312/08]. Italy: Comitato Etico Provinciale Treviso [XLIV-RSA/AULSS7]. The Netherlands: Medisch Ethische Toetsingscommissie Vrije Universiteit Amsterdam [2002/141]. Spain: Comité Ético de Investigación Clínica del Hospital Universitario La Paz Madrid [PI-1080]. Sweden: Till forskningsetikkommittén vid Karolinska Instituted Stockholm [00–132]. UK: Hertfordshire Research Ethics Committee [10/H0311/59]).

The project aimed to evaluate the participants once at baseline (between November 2010 and November 2011) and a second time 12–18 months later. During the assessment the participants underwent a clinical examination and were interviewed at home or in a health care centre by trained physicians and nurses using a standardized questionnaire.

### Measures

*Physical function, pain and stiffness of hand OA* were assessed at baseline and 12–18 months later using the three subscales of the AUSCAN (15 items grouped into 3 scales: pain (5 items), stiffness (1 item), and physical function (9 items)) that utilized a 5-point Likert scale (responses ranged from none to extreme; 0 = none, 1 = mild, 2 = moderate, 3 = severe, and 4 = extreme) [1].

*Physical function, pain and stiffness of hip and/or knee OA* were measured at baseline and 12–18 months later using the three subscales of the WOMAC (24 items grouped into 3 scales: pain (5 items), stiffness (2 items), and physical function (17 items)) that utilized a 5-point Likert scale. Hip/knee pain and stiffness were defined as the maximum value of two joints [2, 3].

All the AUSCAN and WOMAC subscales were normalized from 0 to 100; higher scores indicate worse health status [1–3].

*The Grip strength* of both hands at baseline and 12–18 months later was measured twice, using a strain gauge dynamometer and the result (the highest values of the right and left hands were summed and divided by 2) is expressed in kilograms [26].

*Walking speed* was measured by time, registered in seconds, for a 3-m course marked out on the floor with no obstructions for an additional 2 ft at both ends.

*Anxiety and depression* were evaluated at baseline and 12–18 months later using the Hospital Anxiety and Depression Scales (HADS), a 14-item self-assessment instrument that measures anxiety and/or depression separately [27]. Scores that are 8 or higher (scores range between 0 and 21) on each/either of the subscales indicate altered states.

*Health-related QoL* was measured at baseline and 12–18 months later using: the 5-level EQ-5D, consisting of a descriptive system comprising five dimensions (mobility, self care, usual activities, pain/discomfort, anxiety/depression) and the EQ VAS, a vertical visual analogue scale [28]. The scores of the EQ-5D were converted into a single index, based on general population surveys, using the time trade-off (TTO) valuations from the general population of the UK; scores between − 0.594 and 1. 1 indicate good or satisfying health. Scores of the EQ-VAS range between 0 and 100, with higher scores indicating better health.

*Clinical diagnosis of OA* was formulated on the basis of the participant’s medical history and a physical examination (only at baseline), utilising algorithms in accordance with the clinical criteria developed by the American College of Rheumatology (ACR) [29] and the recommendations of the European League Against Rheumatism [30].

Clinical hand OA (classified as present vs absent) was diagnosed using specific AUSCAN sections [1]: the cut-off in the algorithm for hand pain was ≥3 and it was ≥1 for stiffness. At least 2 of the following criteria were required for a diagnosis of hand OA: a) hard tissue enlargement of two or more joints, b) hard tissue enlargement of two or more distal inter-phalangeal joints, c) deformity of at least one hand joint. Swelling of the metacarpophalangeal joints criteria was a variable that was assessed only in the English and German participants.

Clinical hip/knee OA, defined as the presence of OA in at least one or both of these joints, was diagnosed on the basis of the outcome of specific WOMAC sections. Pain in the hip/knee on at least one side [2, 3] was evaluated during the physical examination using a cut-off ≥3. For the participants, to be diagnosed with knee OA, at least two of the following were necessary: a) a morning stiffness score from mild to extreme; b) crepitus with active motion on at least one side at the physical examination; c) bone tenderness on at least one side at the physical examination; d) bone enlargement at the physical examination on at least one side; e) no palpable warmth of synovium at the physical examination in either knees. All of the following were needed for a positive hip OA assessment: a) pain in the hip on at least one side associated with restricted hip internal rotation at a physical examination; b) morning stiffness of the hip lasting < 60 min, evaluated using the stiffness section of the WOMAC with a score from mild to extreme.

### Statistical analysis

Data analyses and graphical presentations were carried out using SAS software (SAS System, SAS Institute Inc., Cary, NC), version 9.4. Data were analysed using a set of weights calculated per sex and per 5-year age class with respect to the 2010 Standard European Population [4].

The changes over times (in the 12–18 months between the baseline evaluation and the follow-up one) were evaluated as continuous variables using the non-parametric signed rank test. Spearman’s correlation was used to compare the changes in the AUSCAN and WOMAC physical function scales and the changes in the other variables; the Cronbach α coefficient was used to measure the scales’ reliability (internal consistency) (values of α ≥ 0.7 reflect a good reliability) [31].

Only the data of the participants whose assessments were considered complete, that is they had completed both the baseline and follow-up assessments, were included in the statistical analysis. The MCID was calculated by measuring the changes from basal to follow-up measurements scores. Since the MCID for subject-reported outcome measures may vary in different populations and depending on the context, as recommended by Revicki et al. [32], we used multiple approaches to estimate the MCID in the AUSCAN and WOMAC physical function scores to triangulate on a single value or on a small range of values.

For **anchor-based estimation** of MCID we used the receiver operating characteristic (ROC) curve on the change score in the anchor. The variables assessed as possible anchors for the AUSCAN hand OA physical function score were: the AUSCAN for hand OA Pain, the AUSCAN for hand OA Stiffness, the Grip strength, the HADS anxiety, the HADS depression, the EQ-5D-5 L, and the EQ VAS. The variables evaluated as possible anchors for the WOMAC for hip/knee OA physical function score were: the WOMAC for hip/knee OA Pain, the WOMAC for hip/knee OA Stiffness, the Walking-test time, the HADS anxiety, the HADS depression, the EQ-5D-5 L, and the EQ VAS.

An anchor should be chosen because of a significant correlation between the change in the physical function score and the change in the anchor and a correlation coefficient ≥ |0.30| [31].

A ROC curve was constructed for those participants showing stable or worsened anchor scores; the area under the curve (AUC) summarizes the instrument’s ability to distinguish between individuals who have or do not have a minimal clinically important difference in functionality. The criteria used to calculate the probability of an optimal cut-off were: the Youden index (J) [33], the Euclidean distance (D), and the equality sensitivity and specificity (S). The percentage of participants exceeding the MCID were estimated for each cut-off value.

The following were considered for the **distribution based-methods**:

1. A standardized response mean (SRM) [34], representing very small (SRM2), moderate (SRM5) and large changes (SRM8) [35].
2. The standard error of measurement (SEM) [36] of the changes, considering a 63% confidence interval (CI) (SEM63), a 90% CI (SEM90), and a 95% CI (SEM95).
3. The Edwards-Nunnally (EN) method [37], at the 90% CI (EN90), and at the 95% CI (EN95).

Anchor-based and distribution-based-methods were used to determine the MCID, and on that basis the participants were divided into two categories: worse/no worse functionality at the second assessment point. Triangulation was used to examine multiple values from different approaches to converge on a single value, with Cohen’s k (range from − 1 to 1, with one indicating a perfect agreement).

## Results

**Fig. 1:**
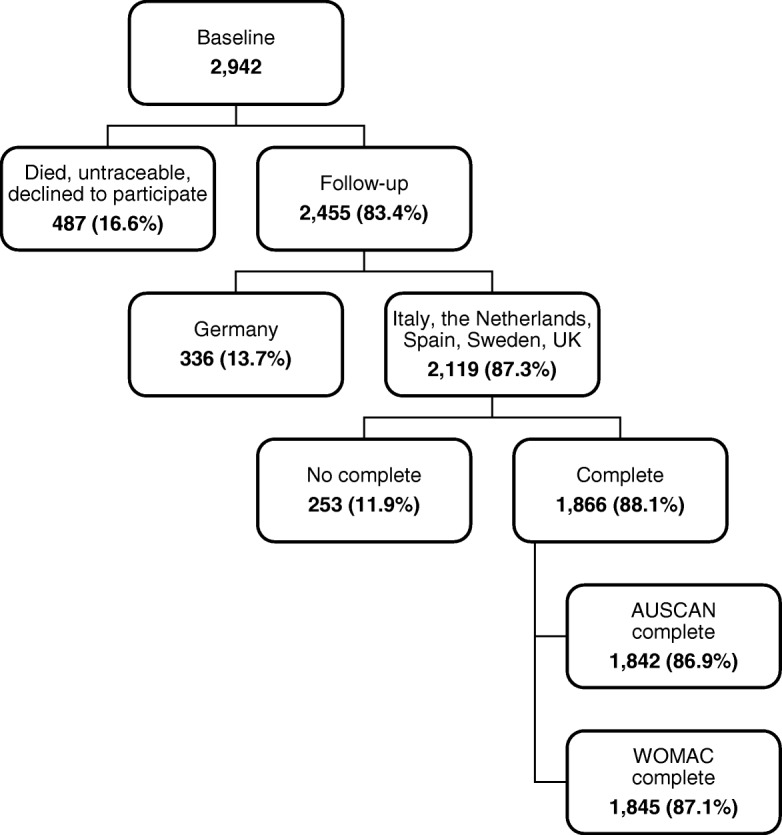
Persons recruited and analysed

Out of the original 2942 participants who completed the baseline evaluation (Fig. 1), 2455 (83%) agreed to undergo a follow-up evaluation 12–18 months later. It was not possible to re-evaluate 487 participants (16.6% of the baseline sample) because they had died, were untraceable, or declined to participate. The non-completers were significantly older, more likely to be female, less educated, and predominantly Italian with respect to the completers.

Since information from the German group was incomplete, data from that country (*n* = 336, 14% of 2455), were not analysed. The data that was analysed and upon which our results are therefore based represents 1866 participants who completed both of the study’s evaluations. One thousand, eight hundred forty-two of these had paired AUSCAN measurements and 1845 had paired WOMAC measurements.

Table 1 (weighted data) shows that approximately 17% of the participants had clinical hand OA and more than 22% had clinical hip/knee OA. The median changes in the physical function scores detected using the AUSCAN hand and WOMAC hip/knee subscales were significant, as were the median changes in the WOMAC stiffness score. There were no significant changes in the AUSCAN pain and stiffness subscales and in the WOMAC pain subscale over time.

**Table 1:** Baseline characteristics and change in physical function and in the anchors after 12–18 months

	Baseline			Change: 12–18 months follow-up - baseline			*P*
Age, n, mean (SD), median (IQR), y	1886	73.8 ± 5.0	73 (70.77)				
Female Sex, n (%)	1886	977 (51.8)					
High education, n (%)	1886	1120 (58.8)					
Country, n (%)	1886						
Italy		312 (16.8)					
The Netherlands		407 (22.0)					
Spain		413 (22.4)					
Sweden		392 (23.0)					
UK		362 (15.8)					
Clinical hand OA, n (%)	1886	1842 (16.7)					
Clinical hip/knee OA, n (%)	1886	1845 (22.6)					
AUSCAN for hand OA^a^, n, mean ± SD, median (IQR)							
Physical function	1842	9.1 ± 15.4	0 (0,11)	1842	1.1 ± 11.3	0 (0,4)	**<.0001**
Pain^c^	1842	7.6 ± 15.2	0 (0,5)	1842	1.7 ± 14.4	0 (0,0)	0.1949
Stiffness	1842	10.0 ± 18.7	0 (0,25)	1842	1.0 ± 17.2	0 (0,0)	0.1358
WOMAC for hip/knee OA^a^, n, mean ± SD, median (IQR)							
Physical function	1845	8.5 ± 13.2	2 (0,12)	1845	1.5 ± 11.3	0 (−1,4)	**0.0123**
Pain	1845	10.0 ± 14.3	5 (0,15)	1845	1.4 ± 13.2	0 (−5,5)	0.2579
Stiffness	1845	12.9 ± 18.6	0 (0,25)	1845	0.3 ± 17.9	0 (0,12)	**0.0390**
Grip strength^b^, n, mean ± SD, median (IQR), kg	1862	27.7 ± 10.2	26 (20,35)	1826	−1.1 ± 5.8	−1 (−3.5,1.5)	**0.0004**
Walking-test time^c^, n, mean ± SD, median (IQR), sec	1854	3.3 ± 1.6	3 (2.3,3.8)	1830	0.1 ± 1.6	0 (−0.5,0.6)	**<.0001**
HADS anxiety^d^, n, mean ± SD, median (IQR)	1886	4.5 ± 3.5	4 (2,7)	1872	−0.6 ± 2.6	−1 (−2,1)	**<.0001**
HADS depression^d^, n, mean ± SD, median (IQR)	1886	3.5 ± 3.0	3 (1,5)	1875	−0.3 ± 2.4	0 (−1,1)	**<.0001**
EQ-5D-5 L^e^, n, mean ± SD, median (IQR)	1876	0.8 ± 0.2	0.8 (0.7,1.0)	1855	0.0 ± 0.2	0 (0.0,0.1)	**0.0059**
EQ VAS^f^, n, mean ± SD, median (IQR)	1882	76.5 ± 16.2	80 (70,90)	1875	−0.5 ± 15.6	0 (−10,5)	**0.0114**

Weighted data. Numbers of subjects, age, and sex were unweighted data. Except where indicated otherwise, values are the percent of subjects

*EPOSA* European Project on OSteoArthritis, *SD* Standard deviation, *IQR* interquartile range, *AUSCAN* AUStralian/CANadian Osteoarthritis Hand Index, *WOMAC* Western Ontario and McMaster Universities Osteoarthritis Index, *OA* osteoarthritis, *HADS* Hospital Anxiety and Depression Scales, *EQ-5D-5 L* Health status using five dimensions, *EQ VAS* Health status using the visual analogue scale

^a^ Possible scores range from 0 to 100, with 100 indicating worse health status. ^b^ Lower values indicate worse performance. ^c^ Higher values indicate worse performance. ^d^ Possible scores range from 0 to 21, a score ≥ 8 indicates anxiety and/or depression. ^e^ Possible scores range from − 0.594 to 1, lower values indicating worse health status. ^f^ Possible scores range from 0 to 100, with 0 indicating worse health status

The *P* are bold where they are less than or equal to the significance level cut-off of 0.05

All other changes in grip strength, walking-test time, the HADS scales, and the EQ-5D-5 L were significant over time.

Table 2 shows the correlation coefficient in the change in the AUSCAN and the WOMAC physical function scores and in the change in the other measures that were considered as possible anchors. For the hand, only the changes in the AUSCAN pain scores were significantly correlated with a coefficient greater than |0.3| (*r* = 0.31) with the changes in the AUSCAN physical function scores. For the hip/knee, both the changes in the WOMAC pain scores and the changes in the WOMAC stiffness scores were correlated with the changes in the WOMAC physical function score with respectively a correlation coefficient of 0.47 and 0.35 (greater than |0.3|).

**Table 2:** Correlation among change in physical function with change in the anchors

Change	AUSCAN for hand OA Physical function (*n* = 1842)		WOMAC for hip/knee OA Physical function (*n* = 1845)	
Anchors	r	*P*	r	*P*
AUSCAN for hand OA Pain	0.30742	*<.0001*		
AUSCAN for hand OA Stiffness	0.21075	*<.0001*		
WOMAC for hip/knee OA Pain			0.46557	*<.0001*
WOMAC for hip/knee OA Stiffness			0.34937	*<.0001*
Grip strength	−0,10,451	*<.0001*		
Walking-test time			0.06917	*0.0034*
HADS anxiety	0.02249	*0.3363*	0.11065	*<.0001*
HADS depression	0.04346	*0.0630*	0.12391	*<.0001*
EQ-5D-5 L	−0.11449	*<.0001*	−0.15293	*<.0001*
EQ VAS	−0.07177	*0.0021*	−0.10105	*<.0001*

*AUSCAN* AUStralian/CANadian Osteoarthritis Hand Index, *WOMAC* Western Ontario and McMaster Universities Osteoarthritis Index, *OA* osteoarthritis, *HADS* Hospital Anxiety and Depression Scales, *EQ-5D-5 L* health status using five dimensions, *EQ VAS* health status using the visual analogue scale, *r* Spearman’s correlation coefficient

The AUSCAN and WOMAC physical function scores showed a very good reliability of coefficient (Cronbach’s α of 0.92 and 0.94 respectively).

### AUSCAN physical function estimates of MCID

Using hand pain as an external anchor, the estimates of the MCID in the AUSCAN hand physical function were consistent and equal to one. The only divergent criteria was the Youden index according to which the estimated MCID for the hand was four. Using distribution-based methods, the estimate for significant worsening in the AUSCAN physical function score ranged from 1 to 8.

**Fig. 2:**
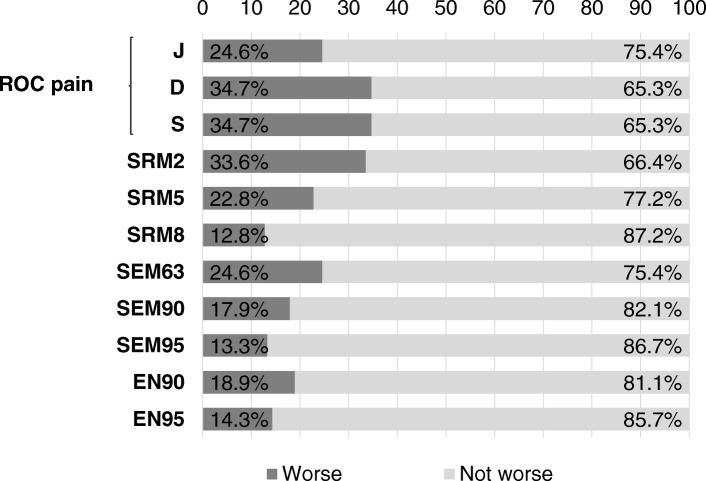
Estimates of MCID percentages for the AUSCAN hand physical function. AUSCAN: Australian/Canadian Hand Osteoarthritis Index; MCID: Minimum Clinically Important Difference; ROC pain: receiver operating characteristic using the AUSCAN pain score as the anchor; J: point that maximizes the Youden index; D: point that minimizes the Euclidean distance; S: point that minimizes the equality sensitivity, specificity. SRM: standardized response mean; SRM2: SRM with Cohen’s threshold 0.20; SRM5: SRM with Cohen’s threshold 0.50; SRM8: SRM with Cohen’s threshold 0.80; CI: Confidence Interval; SEM: Standard Error Measurement; SEM63: SEM with 63% CI; SEM90: SEM with 90% CI; SEM95: SEM with 95% CI; EN: Edwards-Nunnally index; EN90: EN with 90% CI; EN95: EN with 95% CI

Based on these cut-offs, the participants were divided into worse vs not worse in functionality 12–18 months after baseline (Fig. 2).

When the percentage of values obtained with distribution-based MCID methods were compared with those produced by anchor-based methods, the two sets agreed most strongly according to Cohen’s k. The first set (k values at approximately 0.97) formed by the ROC D Euclidean distance, the ROC S for equal sensitivity and specificity, and the SRM2, identified approximately 34–35% of the participants with clinically significant physical function decline at the 12–18 month follow-up evaluation. The second set (k values ranging from 0.95 to 1) which was formed by the ROC J Youden index, the SRM5, and the SEM63, uncovered that 24% of the participants had a clinically significant decline. In view of the concordance and the recommendation to privilege the anchor-based methods [32], we compared the MCID based on the ROC J/SEM63 and the one based on the ROC D/S. Out of the 639 worse participants identified by the ROC D/S criterion, 453 were the same ones identified by ROC J/SEM63. The MCID based on the ROC J/SEM63, which estimated a change of 4 points, was found to be the most reliable criterion to analyse the loss of hand functionality at 12–18 months.

### WOMAC physical function estimates of MCID

Using hip/knee pain and stiffness scales as external anchors, the estimates of the MCID of the WOMAC hip/knee physical function were consistent, although the magnitude of the correlations can only be considered moderate. The ROC analysis of the anchor responses to the WOMAC pain and stiffness scales estimated that the MCID was almost always one. Once again, the divergent criteria was the Youden index with stiffness as the anchor that estimated a two point MCID for hip/knee physical function.

**Fig. 3:**
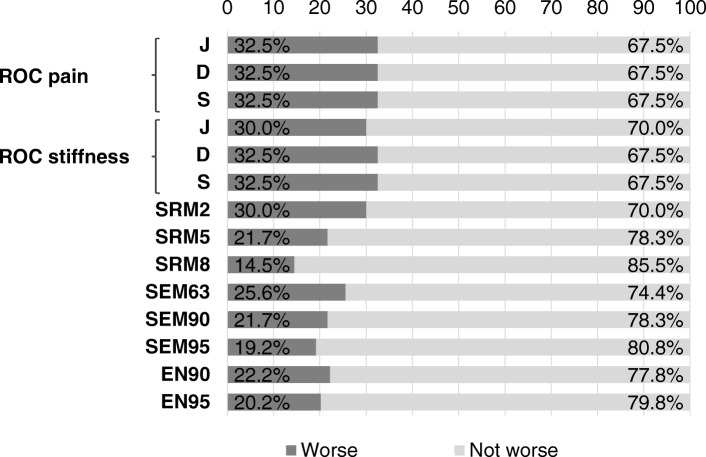
Estimates of MCID percentages for the WOMAC hip/knee physical function. WOMAC, Western Ontario and McMaster Universities; MCID: Minimum Clinically Important Difference; ROC pain: receiver operating characteristic using the WOMAC pain score as the anchor; ROC stiffness: receiver operating characteristic using the WOMAC stiffness score as the anchor; J: point that maximizes the Youden index; D: point that minimizes the Euclidean distance; S: point that minimizes the equality sensitivity, specificity. SRM: standardized response mean; SRM2: SRM with Cohen’s threshold 0.20; SRM5: SRM with Cohen’s threshold 0.50; SRM8: SRM with Cohen’s threshold 0.80; CI: Confidence Interval; SEM: Standard Error Measurement; SEM63: SEM with 63% CI; SEM90: SEM with 90% CI; SEM95: SEM with 95% CI; EN: Edwards-Nunnally index; EN90: EN with 90% CI; EN95: EN with 95% CI

Using distribution-based methods, the estimate for significant worsening in the WOMAC physical function score ranges from 1 to 9. Figure 3 shows the percentage of participants who had worse hip/knee functionality 12–18 months after baseline according to the different methods utilized. The MCIDs for hip/knee physical function decline that showed the highest degree of agreement were: those based on the SRM2, those that used the WOMAC pain score as the anchor minimized the Euclidean distance and the equality sensitivity and specificity as well as those that maximized the Youden index (*k* = 0.94). The SRM2 also agreed with those that, using the WOMAC stiffness score as the anchor, minimized the Euclidean distance and the equality sensitivity and specificity (*k* = 0.94), or maximized the Youden index (*k* = 1). These methods identified 30 and 33% of participants with clinically significant hip/knee physical function decline 12–18 months after baseline respectively. Finally, there was a strong agreement (*k* = 0.89) between the SEM63 and the Youden index with the stiffness score used as an anchor; they respectively detected 26 and 30%.of the participants.

As the highest degree of agreement was found between the Youden index (using ROC with stiffness as the anchor) and the SRM2, the MCID based on these criteria seemed to be the most suitable one to analyse the loss of hip/knee functionality at 12–18 months. Both criteria were consistent in identifying two as the best discriminating WOMAC physical function change cut-off.

## Discussion

While it is true that the AUSCAN and WOMAC scales are the most commonly used clinical tools to manage and monitor OA patients, to our knowledge the MCID for decline picked up by these measures has never been evaluated. Only the study of Angst et al. [9], which focused on patients with OA of the lower extremities after a rehabilitation intervention, reported a MCID showing worsening in the WOMAC hip/knee physical function of approximately 1.33, based on a scale from 0 to 10. The study’s initial premise that hand and hip/knee physical function would deteriorate significantly over a year’s time was confirmed by our data showing higher AUSCAN and WOMAC physical function scores.

Although the relevance of the MCID approach remains controversial and despite the fact that physical function values can depend on the population being examined, the context, the time and methods used [25], it remains an important assessment instrument. The magnitude of the MCID was inferior in the participants studied here using the WOMAC instrument with respect to other studies [7–15]. As those studies focused on patients before and after interventions, the differences in the magnitude of the MCID might be connected to patient expectations regarding surgical interventions, as compared to non-surgical interventions [38]. Other factors that might explain the differing MCID values could be: the severity of the participant’s baseline health status, the length of the period being examined, the accuracy of the measurement instruments, and the direction in the change in the MCID (i.e. towards improvement or worsening).

Other studies have demonstrated that changes in the AUSCAN and WOMAC physical function scores correlate significantly with changes in other generic, adapted, or performance-based measures used to gauge pain and function in the hand and hip/knee OA [18–25]. Our study did not, however, find any correlations between the changes in the AUSCAN and WOMAC physical function and the changes in other more generic measures such as the Hospital Anxiety and Depression Scale and the European QoL Surveys. The anchors with the strongest correlations were the pain-specific questionnaires (AUSCAN and WOMAC pain subscale), presumably because they are basically measures of pain during physical activities rather than unspecific pain measures [39].

The estimates of the MCID in both the AUSCAN hand physical function and the WOMAC hip/knee physical function according to the ROC analysis using different anchor responses and criteria, were consistent. The only divergent criteria was the Youden index that overestimated the MCIDs.

But as explained above, besides an anchor-based method, we also used a distribution-based approach to estimate the MCID, given that the two are complementary [40]. Distribution-based approaches, which are based on the statistical characteristics of the samples studied and reliable measures, generated a wide range of different estimates of the MCID for the AUSCAN hand and WOMAC hip/knee physical function that were greater than the anchor-based estimates. Both methods converged to a common result.

While distribution-based estimates are able to furnish supportive information when the change is significant, they do not provide a direct measure of minimum clinically important difference. That is why precedence was shown to the anchor based estimates. Moreover, since the MCIDs estimated using distribution-based methods were greater than the mean change reported 12–18 months after baseline, it is possible that the data from the distribution-based methods provide information about clinical significance but might overestimate the true MCID.

This study has several limitations. First, the changes in outcome measures could hypothetically be associated with baseline levels. Second, there is the possibility that the participants selected did not experience much or any change over the 12–18 month study period. Third, there may be even important differences in the populations studied and in the cut-off values of the MCID physical function decline. Indeed, estimates can differ depending on the instrument, domain, country, and condition, at least for condition-specific measures, and further research is required before the estimates presented here can be generalized to other instruments [7].

The study’s most important strength was undeniably its large population base: the participants were randomly selected from older community-dwelling European populations. Not only persons with OA but also large numbers of healthy individuals not affected with OA were analysed. The methodology used was the same in all of the countries, and OA was diagnosed in accordance with standardized international guidelines [4]. Our study was based on valid standardized globalized measures (the WOMAC and AUSCAN Indexes) suggested by guidance documents [41, 42], and, in fact, they proved to be quite reliable. The data are longitudinal in nature. Another study strength was that it describes the decision-making process leading to the selection of a single value from a range of different MCID cut offs by comparing the percentages of change scores exceeding the MCID. The process considered the major concordance between those based on anchor-based methods and those based on distribution-based approaches, privileging those based on the former [40, 43, 44], and evaluated the differences in terms of clinical OA.

## Conclusion

To conclude, the study shows that the AUSCAN hand and WOMAC hip/knee physical function scores are indeed sensitive to the effects of OA. The data analysed using various health and physical performance measures as external anchors showed that the minimally important decline over 1 year in the AUSCAN and WOMAC physical function scores was four and two points respectively. Further research is required to confirm the robustness of these estimates and to evaluate their temporal consistency and country-dependency.

## Abbreviation

AUSCAN: Australian/Canadian Hand Osteoarthritis hand Index
CI: Confidence Interval
EN: Edwards-Nunnally index
EN90: EN with 90% CI
EN95: EN with 95% CI
EPOSA: European Project on OSteoArthritis
EQ VAS: Health status using the visual analogue scale
EQ-5D-5 L: Health status using five dimensions
HADS: Hospital Anxiety and Depression Scales
IQR: interquartile range
MCID: Minimum Clinically Important Difference
ROC: Receiver operating characteristic
SD: Standard deviation
SEM: Standard Error Measurement
SEM63: SEM with 63% CI
SEM90: SEM with 90% CI
SEM95: SEM with 95% CI
SRM: standardized response mean
SRM2: SRM with Cohen’s threshold 0.20
SRM5: SRM with Cohen’s threshold 0.50
SRM8: SRM with Cohen’s threshold 0.80
WOMAC: Western Ontario and McMaster Universities knee and hip Index
